# T118M Variant of PMP22 Gene Presents with Painful Peripheral Neuropathy and Varying Charcot-Marie-Tooth Features: A Case Series and Review of the Literature

**DOI:** 10.1155/2018/2618071

**Published:** 2018-12-25

**Authors:** Kwo Wei David Ho, Nivedita U. Jerath

**Affiliations:** University of Florida, Department of Neurology, USA

## Abstract

The clinical effect of T118M variant of the PMP22 gene has been controversial. Several studies have suggested that it may be autosomal recessive, partial loss of function, or a benign variant. Here we report three cases in further support that the T118M variant of the PMP22 gene is a partial loss of function variant. These three unrelated cases were heterozygotes with the T118M variant of the PMP22 gene. All three cases presented with painful peripheral neuropathy and varying degrees of Charcot-Marie-Tooth exam features. Electrophysiological studies revealed polyneuropathy with axonal and demyelinating features in one case, but there were minimal electrophysiological changes in the other two cases. We propose that the T118M variant can cause painful peripheral neuropathy, which may be an underrecognized feature of this variant.

## 1. Introduction

PMP22 is a myelin membrane protein expressed in Schwann cells. Missense mutations in PMP22 cause a variety of neuropathies, including Charcot-Marie-Tooth disease (CMT)[1] and hereditary neuropathy with liability to pressure palsies (HNPP). The majority of these mutations act in autosomal dominant pattern [2–5], and a few mutations are autosomal recessive in nature [2, 6, 7]. A missense mutation at codon 118 (T118M) in PMP22 has been reported in several reports in the context of familial neuropathy. However, the clinical relevance of the T118M variant of the PMP22 gene has been controversial. Several studies have suggested that it may be autosomal recessive [2], partial loss of function [8–10], or a benign variant [11]. In this study, we report three cases in support that the T118M variant of the PMP22 gene is a partial loss of function mutation rather than a benign variant. We propose that the T118M variant can cause painful peripheral neuropathy and varying degrees of Charcot-Marie-Tooth features.

## 2. Methods

### 2.1. Genetic Testing

All subjects received care at the neurology clinic at the University of Florida. Genetic testing was performed by Invitae Co. (San Francisco, CA). Genomic DNA obtained from the submitted sample is enriched for targeted regions using a hybridization-based protocol and sequenced using Illumina technology. Unless otherwise indicated, all targeted regions are sequenced with ≥50x depth or are supplemented with additional analysis. Reads are aligned to a reference sequence (GRCh37), and sequence changes are identified and interpreted in the context of a single clinically relevant transcript, indicated below. Enrichment and analysis focus on the coding sequence of the indicated transcripts, 10bp of flanking intronic sequence (20bp for BRCA1/2), and other specific genomic regions demonstrated to be causative of disease at the time of assay design. Promoters, untranslated regions, and other noncoding regions are not otherwise interrogated. Exonic deletions and duplications are called using an in-house algorithm that determines copy number at each target by comparing the read depth for each target in the proband sequence with both mean read-depth and read-depth distribution, obtained from a set of clinical samples. All clinically significant observations are confirmed by orthogonal technologies, except individually validated variants and variants previously confirmed in a first-degree relative. Confirmation technologies include any of the following: Sanger sequencing, Pacific Biosciences SMRT sequencing, MLPA, MLPA-seq, or Array CGH.

The following transcripts were used in this analysis: AARS (NM_001605.2), AIFM1 (NM_004208.3), ATL1 (NM_015915.4), ATL3 (NM_015459.4), ATP7A (NM_000052.6), BICD2 (NM_001003800.1), BSCL2 (NM_032667.6), CHCHD10 (NM_213720.2), DCTN1 (NM_004082.4), DNAJB2 (NM_001039550.1), DNM2 (NM_001005360.2), DNMT1 (NM_001130823.1), DST (NM_001723.5), DYNC1H1 (NM_001376.4), EGR2 (NM_000399.3), FAM134B (NM_001034850.2), FBXO38 (NM_030793.4), FGD4 (NM_139241.3), FIG4 (NM_014845.5), GAN (NM_022041.3), GARS (NM_002047.2), GDAP1 (NM_018972.2), GJB1 (NM_000166.5), GNB4 (NM_021629.3), HARS (NM_002109.5), HINT1 (NM_005340.6), HSPB1 (NM_001540.3), HSPB8 (NM_014365.2), IGHMBP2 (NM_002180.2), IKBKAP (NM_003640.3), INF2 (NM_022489.3), KIF1A (NM_004321.6), LITAF (NM_004862.3), LMNA (NM_170707.3), LRSAM1 (NM_138361.5), MED25 (NM_030973.3), MFN2 (NM_014874.3), MORC2 (NM_014941.2), MPZ (NM_000530.6), MTMR2 (NM_016156.5), NDRG1 (NM_006096.3), NEFL (NM_006158.4), NGF (NM_002506.2), NTRK1 (NM_001012331.1), PDK3 (NM_001142386.2), PLEKHG5 (NM_020631.4), PMP22 (NM_000304.3), PRPS1 (NM_002764.3), PRX (NM_181882.2), RAB7A (NM_004637.5), REEP1 (NM_022912.2), SBF2 (NM_030962.3), SCN11A (NM_014139.2), SCN9A (NM_002977.3), SH3TC2 (NM_024577.3), SIGMAR1 (NM_005866.3), SLC25A46 (NM_138773.2), SLC52A2 (NM_024531.4), SLC52A3 (NM_033409.3), SLC5A7 (NM_021815.2), SPG11 (NM_025137.3), SPTLC1 (NM_006415.3), SPTLC2 (NM_004863.3), TFG (NM_006070.5), TRIM2 (NM_001130067.1), TRPV4 (NM_021625.4), TTR (NM_000371.3), UBA1 (NM_003334.3), VAPB (NM_004738.4), VRK1 (NM_003384.2), WNK1 (NM_018979.3), YARS (NM_003680.3).

### 2.2. Literature Review

We performed a literature review on the published clinical studies on T118M variant in the PMP22 gene. The following terms were used to search in Pubmed and Google Scholar: “T118M” and “PMP22”. We restricted our review to published full articles since 1950 in the English language. We excluded basic science research articles that did not present new patients with the variant.

## 3. Clinical Report

### 3.1. Case 1

The first patient was a 64-year-old male presenting with painful burning pain at the bottom of his feet for six months. The pain extended from his feet up to his legs, hips, and back in a sharp shooting manner. It was constant and it was so severe that it limited his activities. As a child, he started walking later than his peers and he was always the slowest runner. He used leg braces because his knees were “together” and he had a surgery for it at age 15. He had occasional muscle cramps and fasciculations as a child. His mother was always clumsy in her feet as well. He had no siblings or children. He had a CMT examination score of 8 out of 28. On exam, there was pes cavus bilaterally and tight Achilles tendons. His feet could not be easily brought into a neutral position. There was atrophy of the hands and feet, length-dependent pinprick and vibratory sense loss, and absent reflexes. MRI of the lumbar spine was unremarkable. Electrophysiological studies revealed moderate chronic sensorimotor, axonal polyneuropathy (Table 1). There were absent sensory responses in the bilateral sural and superficial peroneal nerves. Motor studies showed reduced amplitude in the left tibial nerve and reduced conduction velocities ranging from 32-36 m/s in the bilateral peroneal nerves and left tibial nerve. F wave in the bilateral peroneal nerve showed prolonged latency. F wave in the bilateral tibial nerve was absent. Sequencing of 72 neuropathy genes [15] showed one copy of a pathogenic variant, T118M in the PMP22 gene.

### 3.2. Case 2

**Figure 1:**
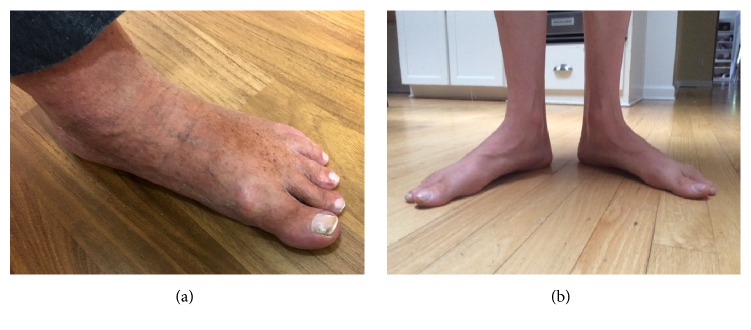
Flat feet with low arches in case 2 (a) and case 3 (b).

The second patient was a 73-year-old man from Cuba presenting with leg pain which he described as a constant burning pain in his feet and aching pain in his legs. He was never a fast runner as a child and he was not athletic. His sister had similar symptoms of flat and painful feet. Exam revealed flat feet (Figure 1(a)), absent reflexes, and absent vibratory sense at the toes and reduced at the ankles. Electrophysiological study was unremarkable except for a mildly reduced peroneal nerve conduction velocity at the fibular head (Table 1). CMT examination score was 6 out of 28. Genetic testing revealed heterozygous T118M variant of the PMP22 gene and heterozygous R275L variant of the SLC52A2 gene. The sister was unable to undergo genetic testing.

### 3.3. Case 3

The third patient was a 56-year-old male with past medical history of Sjögren's syndrome and rheumatoid arthritis who presented with chronic severe burning pain in the hands and feet necessitating the chronic use of narcotics to allow him to continue his profession. As a child he did have some difficulties with coordination and playing basketball. He had a daughter who also had flat feet and not athletic. He did not have other siblings. Exam showed decreased vibratory sense in the toes and flat feet with low arches (Figure 1(b)). Reflexes were present. CMT examination score was 2 out of 28. Electrophysiological study was unremarkable except for a mildly reduced tibial motor conduction velocity at the popliteal fossa (Table 1). Skin biopsy of the right distal leg and proximal thigh revealed normal epidermal small fiber densities. Sjögren's syndrome profile showed positive salivary protein IgA antibodies, parotid specific protein IgG, IgA, IgM antibodies, positive rheumatoid factor, and anticyclic citrullinated peptide antibody. ANA, double stranded DNA antibody, TSH, and free T4 were negative. Genetic testing showed heterozygous T118M variant of the PMP22 gene and heterozygous Y22C variant of the TFG gene (c.98 A> G). The daughter declined genetic testing.

## 4. Literature Review

The result of the literature review is presented in Table 2. Eight studies have been published on the clinical impact of T118M variant in the PMP22 gene. There was one retrospective case-control study, six family case series, and one case report. Three small family case series introduced the notion that the T118M variant is either a benign polymorphism or an autosomal recessive mutation [2, 10, 12]. However, other studies presented data in support of it being a deleterious variant. The largest study is a case-control study with 1018 healthy subjects, 104 unrelated patients with hereditary neuropathy with liability to pressure palsies (HNPP), and 187 patients with Charcot-Marie-Tooth disease type 1 (CMT1) [11]. It found T118M to be associated with CMT1A without the 1.5-Mb duplication (P=0.0429), but not associated with HNPP or CMT1 with the 1.5Mb duplication. The allele frequency of T118M was much higher in CMT1 without duplication (AF=0.05) compared to the general population (AF=0.007). One case series [8] and one case report [14] gave further support to the notion that the T118M is rather a loss of function variant to cause varying degrees of neuropathies. Shy et al. [8] reported the first homozygous case presenting with severe axonal neuropathy, while the heterozygotes in the same study presented with milder form of neuropathy similar to HNPP.

## 5. Discussion

In this case series, we demonstrated that patients with the T118M variant of the PMP22 gene can present with peripheral neuropathic pain and varying features of CMT. None of the cases presented here harbor the typical 1.5-Mb deletion in 17p11.2 seen in HNPP, or the typical duplication of the same locus in CMT1. This study supports the notion that the T118M variant of the PMP22 gene can be a partial loss of function variant to possibly lead to a disease state marked by painful peripheral neuropathy and certain CMT features.

The T118M variant, or rs104894619, is a rare variant having a minor allele frequency of 0.0008 in the 1000 genome project [16]. Several previous studies have suggested that the T118M variant can be a benign polymorphism (Table 1). However, Shy et al. refuted such a notion with a patient homozygous for this variant, and this patient presented with severe axonal neuropathy [8]. This study also found varying degrees of clinical and electrophysiological features of a neuropathy similar to HNPP in heterozygous patients and they concluded that the T118M variant is a loss of function mutation. The largest case control study [11] also suggests that this variant is associated with CMT1 in the absence of the typical PMP22 duplication. Our report adds to the literature by proposing that painful peripheral neuropathy can be a feature of this variant as well.

The T118M variant has been shown to cause cellular disruptions in several in vitro studies. When PMP22 with the T118M allele was expressed in the absence of wild-type PMP22, the apoptotic-like phenotype of the NIH-3T3 cells was reduced. Coexpression of the T118M-PMP22 with the wild-type PMP22 restored the apoptotic phenotype [17]. In another study, the T118M allele has altered intracellular trafficking compared to the wild type [18]. It is therefore not surprising that the T118M variant can produce an abnormal phenotype.

Despite the common T118M variant among the three cases, electrophysiological findings were quite varied. Case 1 presented with severe sensorimotor polyneuropathy with both axonal and demyelinating features, while cases 2 and 3 presented with mild reduction in conduction velocities. It is unclear why case 2 had decreased vibratory sense loss, absent reflexes but only mildly abnormal electrophysiological study. It has been reported that electrographically recorded deep tendon reflex can be present even in the absence of clinical deep tendon reflex [19]. The varied EMG findings are consistent with previous findings that patients carrying this variant can have electrophysiological findings ranging from mildly prolonged latency to severe axonal neuropathy [8]. This study is in further support of such varied presentation.

Genetic factors are known to impact pain perception and formation. Common genetic polymorphisms have been shown to affect the development and perception of pain [20, 21]. Rare, single-gene variants causing painful phenotype are less common [22–24]. Painful peripheral neuropathy is a common feature in both CMT [1] and HNPP [25]. However, peripheral neuropathic pain is often overlooked due to other predominant features in CMT and HNPP. In patients presenting with an isolated painful neuropathy without many CMT or HNPP features, genetic testing is rarely performed. Given the findings in this case series, the T118M variant of the PMP22 gene can be a possible cause of painful neuropathy even without significant nerve conduction abnormalities. With genetic testing becoming more readily available, genetic variants such as T118M in PMP22 should be considered in patients presenting with idiopathic painful peripheral neuropathy.

The skin biopsy of case 3 showed normal epidermal small fiber densities. The significance of this finding is unclear. The most likely explanation is that the biopsied sites (leg and thigh) were unaffected by the disease process, given that the symptomatic sites were hands and feet. An alternative explanation may be that the variant does not affect epidermal nerve fiber density in mild cases. In addition to the normal skin biopsy, case 3 also had Sjögren's syndrome and rheumatoid arthritis. Both Sjögren's syndrome and rheumatoid arthritis are known to cause peripheral neuropathy [26, 27]. It is unclear if these rheumatological factors contributed to this patient's painful polyneuropathy.

Two of the three cases presented here were tested positive for genetic variants other than T118M in PMP22. One of them was heterozygous for R275L variant of the SLC52A2 gene and one of them was heterozygous for Y22C variant of the TFG gene. Both individuals had minimal electrophysiological changes. Variants in SLC52A2 gene have been found to be associated with autosomal recessive Brown-Vialetto-Van Laere syndrome, which is characterized by infancy onset sensorineural deafness and pontobulbar palsy. The R275L variant has not been reported to be associated with this particular disease and the patient presented here did not exhibit any symptoms characteristic of Brown-Vialetto-Van Laere syndrome. A variant in TFG (p.Gly269Val) has been associated with CMT2 phenotype. However, Y22C has not been reported to cause any CMT2 phenotype and its significance is currently unknown.

In summary, we demonstrated that the T118M variant of the PMP22 gene can present with peripheral neuropathic pain and features of CMT. This case series supports the argument that the T118M variant of the PMP22 gene can be a partial loss of function mutation to possibly lead to a disease state marked by painful peripheral neuropathy.

## Figures and Tables

**Table 1:** Electrophysiological studies of the three cases. Amp: amplitude (*μ*V) and Vel: velocity (m/s). NR: no response. Bold numbers indicate abnormal values.

Response	Nerve	Sites		Case 1	Case 2	Case 3
Sensory response			Genotype (T118M)	+/-	+/-	+/-
			Age	64	73	56
	Left median	Wrist	Amp	-	38.5	-
			Vel	-	53	-
	Left ulnar	Wrist	Amp	-	6.1	-
			Vel	-	43	-
	Left radial	Wrist	Amp	-	27.8	-
			Vel	-	53	-
	Left sural	Calf	Amp	**NR**	6.1	7
			Vel	**NR**	39	52
	Right sural	Calf	Amp	**NR**	7.4	10
			Vel	**NR**	46	46
	Left superficial peroneal nerve	Calf	Amp	**NR**		-
			Vel	**NR**		-
	Right superficial peroneal nerve	Calf	Amp	**NR**	-	-
			Vel	**NR**	-	-

Motor response	Left median nerve	Wrist	Amp	-	11.4	-
		Elbow	Amp	-	10.7	-
			Vel	-	56	-
	Left ulnar	Wrist	Amp	-	14.6	-
		Below elbow	Amp	-	13.4	-
			Vel	-	62	-
		Above elbow	Amp	-	12.2	-
			Vel	-	56	-
	Left peroneal	Ankle	Amp	**2.8**	5.5	5.9
		Fibular head	Amp	**1.6**	4.7	5.4
			Vel	**33**	**39**	43
		Popliteal fossa	Amp	**1.6**	4.7	5.2
			Vel	**32**	48	41
	Left tibial	Ankle	Amp	**0.8**	7.9	7.2
		Popliteal fossa	Amp	**0.3**	4.2	6.8
			Vel	**33**	47	**38**
	Right tibial	Ankle	Amp	**0.9**	-	14.7
		Popliteal fossa	Amp	**0.4**	-	10.2
			Vel	**35**	-	41
	Right peroneal	Ankle	Amp	**4.5**	-	10.4
		Fibular head	Amp	**3**	-	8.4
			Vel	**36**	-	46
		Popliteal fossa	Amp	**2.9**	-	7.9
			Vel	**36**	-	46

**Table 2:** . Summary of clinical studies on T118M mutations of the PMP22 gene.

Author	Year	Study design	Number of subjects	T118M genotype	Findings	Supported modality of disease
Roa et al. [2]	1993	Case series	1 family	Heterozygote	One patient heterozygous for T118M did not show symptoms. One patient hemizygous for both T118M and 1.5-Mb deletion had severe neuropathy.	Benign polymorphism

Nelis et al. [12]	1994	Case series	2 families	Heterozygote	One patient heterozygous for T118M showed CMT1 symptoms, but father showed no symptoms despite having the variant. Another patient with T118M variant in another CMT1 family showed no symptoms.	Benign polymorphism

Mersiyanova et al. [13]	2000	Mutation screen of CMT and HNPP patients.	174 unrelated CMT patients and 3 HNPP families	Heterozygote	Unclear which patient(s) had the T118M variant.	Unclear

Young et al. [11]	2000	Case control	1018 healthy, 104 with HNPP, 187 with CMT1 with 1.5-Mb duplication, 22 with CMT1 phenotype without PMP22 mutations.	Heterozygote	Minor allele frequency lower in general population (AF=0.007) compared to HNPP cases (AF= 0.01), CMT1 with 1.5Mb duplication (AF=0.016) and CMT1 without duplication (AF=0.05). T118M was associated with CMT1A without the 1.5-Mb duplication (P=0.0429), but not with HNPP or CMT1 due to the low allele frequency.	Supports the association of T118M with CMT1A in the absence of the PMP22 duplication.

Marques et al. [9]	2003	Case series	1 family	Heterozygote	Three patients with CMT1 were genotyped. Two had both the 17p11.2-p12 duplication and the T118M variant, while one had only the duplication. Phenotype only available in proband (T118M + duplication). Unclear whether T118M worsened phenotype.	Unclear

Seeman et al. [10]	2006	Case series	1 family	Heterozygote	Two asymptomatic patients had T118M variant, one patient had both the T118M variant and the 17p11.2-12 duplication and had CMT1 phenotype.	Benign polymorphism

Shy et al. [8]	2006	Case series	5 unrelated kindreds	3 with T118M/normal, 2 with T118/deletion, 1 with (T118+duplication)/normal, 2 1 with T118M/T118M	T118M/deletion had severe demyelinating phenotype, T118M/T118M had severe axonal phenotype, T118M/normal had mild HNPP phenotype, (T118M + duplication)/normal had mild demyelinating phenotype	Partial loss of function

Jerath et al. [14]	2015	Case report	1 case	T118M/17p11.2-p12 deletion	Severe sensorimotor polyneuropathy	Benign polymorphism vs partial loss of function

This study	2018	Case series	3 unrelated cases	Heterozygote	All three had painful polyneuropathy. 1 had moderate sensorimotor polyneuropathy with both axonal and demyelinating features, 2 had mildly decreased conduction velocity.	Partial loss of function – can cause painful polyneuropathy
